# Variation in the Concentration of Metals in Road Dust Size Fractions Between 2 µm and 2 mm: Results from Three Metallurgical Centres in Poland

**DOI:** 10.1007/s00244-019-00686-x

**Published:** 2019-11-08

**Authors:** Agata Logiewa, Agnieszka Miazgowicz, Klaus Krennhuber, Christof Lanzerstorfer

**Affiliations:** 1 Faculty of Energy and Environmental Engineering, Silesian University of Technology, Stanisława Konarskiego 18, 44-100 Gliwice, Poland; 2 School of Engineering/Environmental Sciences, University of Applied Sciences Upper Austria, Stelzhamerstraße 23, 4600 Wels, Austria

## Abstract

The composition of road dust is influenced by emissions from local industry as well as by traffic emissions. Thus, the composition of urban road dust can be used as an indicator for environmental pollution. Pollutants contained in road dust also are transferred into the atmosphere by resuspension and into the aquatic system by wash-off. In this transfer, the particle size of the road dust particles is of extreme importance. Therefore, information about the composition of road dust in dependence of the particle size is crucial. In this study, road dust samples were separated by air classification into size fractions down to 2 µm. The chemical analysis of the size fractions also revealed a significant size dependence of the metal concentrations in the finest size fractions. The least polluted size fraction was generally the fraction 200–500 µm, whereas the highest concentrations were measured in the finest size fraction < 2 µm. These results are important for the assessment of the mass fraction of the various pollutants in the mobile size fractions in re-entrainment as well as in run-off during rainfall.

In recent years, numerous studies were carried out in many countries to determine the concentrations of metals in road dust (Adamiec et al. 2016; Ewen et al. 2009; Shi et al. 2008; Apeagyei et al. 2011; Duong and Lee 2011; Bourliva et al. 2017). Emissions from local industry as well as traffic emissions contribute significantly to the composition of road dust. Thus, the composition of urban road dust can be used as an indicator for environmental pollution. Several studies showed a dominant influence of traffic emissions on the composition of road dust (Wrobel et al. 2000). However, close to major heavy industry or mining activities (Ordonez et al. 2003; Žibret et al. 2013), as well as for certain metals (Shi et al. 2010), the importance of the contribution of industrial emissions was demonstrated.

Several studies have shown that resuspended road dust is one of the most important sources of aerosol pollution in large cities (Han et al. 2007; Almeida et al. 2006; Wang et al. 2016), especially in the size range PM 2.5–10 (Pérez et al. 2010). In resuspension of road dust by turbulence and shear stress caused by tires and wind, particles in the size range < 10 µm become more easily resuspended into the atmosphere than coarser particles. These fine particles are transported greater distances, whereas coarse particles are mainly redeposited on the road (Patra et al. 2008). The finest size fractions of road dust represent a major health concern, because after resuspension these fine particles can be readily inhaled and become embedded in human lungs (Wong et al. 2006).

In the removal and transport of road dust by wash-off during rainfall, the particle size is also a key parameter, because the finer particles < 105 µm (Zhao et al. 2016), < 125 µm (Wang et al. 2017), or < 250 µm (Zafra et al. 2017) are removed to a higher degree than the coarser particles (Zhang et al. 2015). Thus, the composition of the fine size fractions of road dust is of increased relevance for the pollution of the run-off.

Because of the environmental relevance, the size dependence of the metal concentrations in road dust has been investigated in several studies. For this purpose, the collected samples were usually separated into various size fractions by dry sieving. Due to the limitations of dry sieving, the finest size fraction in these studies was typically 0–40 µm or larger (Deletic and Orr 2005; Duong et al. 2006; Han et al. 2008; Zafra et al. 2011; Fujiwara et al. 2011; Zhao and Li 2013; Shen et al. 2016). The upper size limit for the road dust investigated in these studies varied between 100 µm and 2 mm. In a few studies, the finest size fraction was smaller. Adamiec et al. (2016) produced five size fractions by sieving where the finest size fraction was 0–20 µm and the coarsest was > 250 µm. In another study, air classification was used for size fractionation of road dust (Lanzerstorfer 2018), producing a finest size fraction with a mass median diameter of approximately 2 µm. However, in this study the upper size limit of the investigated road dust was 200 µm.

All studies on the size dependence of metals in road dust have shown that the concentrations are increased in the finer particles. The reasons for this effect are the larger surface area, surface charge, organic matter content, and metal sorption capacity of these particles (Loganathan et al. 2013).

The purpose of this study was to expand the knowledge about the size dependence of the concentration of metals in road dust to the very fine size fractions. The finer fractions of road dusts were seldom analysed and publicised, although the environmental impact is highly significant, because these fine particles can easily be transported in the atmosphere after resuspension or migrate into the aquatic system by wash-off during rainfall. This was possible by applying air classification for size separation of the collected road dust samples additionally to sieving. The new combined classification method was applied in the investigation of road dust samples collected in three industrial towns in southern Poland with local metallurgical industry and mining activities.

## Materials and Methods

### Study Area

The road dust samples were collected in three industrial and mining towns located in southern Poland: Krakow, Katowice, and Olkusz.

Krakow is the second largest city in Poland with a population of approximately 760,000. The town is situated on the Vistula River in the Lesser Poland region. The road dust sample was collected in Nowa Huta, the easternmost district of Krakow, where the second biggest integrated steel mill in Poland, ArcelorMittal Poland SA, with an annual production of nearly 1.5 million tons of steel, is located.

Katowice is one of the main centres of the Upper Silesian industrial district with a population of more than 300,000. It is an old mining area with three hard coal mines still in operation: KWK Wujek, KWK Wieczorek, and KWK Murcki-Staszic. In Katowice, the heavy industry also plays an important role. Currently four steel mills are located in Katowice: Ferrum SA, BGH Polska (Edelstahl), ZM Silesia, and BATERPOL S.A.

Olkusz is a town located in the Lesser Poland region with a population of approximately 36,000. The town is one of the most important centres of zinc and lead ore mining and processing in Poland. There are both modern industrial plants as well as numerous sites of past exploitation of ores and processing of the metals Zn, Pb, Cd, Ag, Tl, Ge, and Ga. Currently, there is only one zinc and lead ore mine, Pomorzany mine, in operation, which started exploitation in 1975. Two other mines were closed around 2000. Additionally, processing plants and waste disposals accompany mining (Adamczyk et al. 2000).

### Sample Collection and Analytical Procedure

#### Collection of Road Dust Samples

The road dust samples were collected in Spring 2017. Before the sampling, there was an antecedent dry-weather period of at least 3 days. Sampling took place on April 9th in Olkusz, on April 11th in Krakow, and on April 12th in Katowice. The samples from Olkusz and Katowice were collected manually using street sweeper brooms and a shovel, while the sample from Krakow was obtained from a vacuum street sweeping vehicle.

**Fig. 1:**
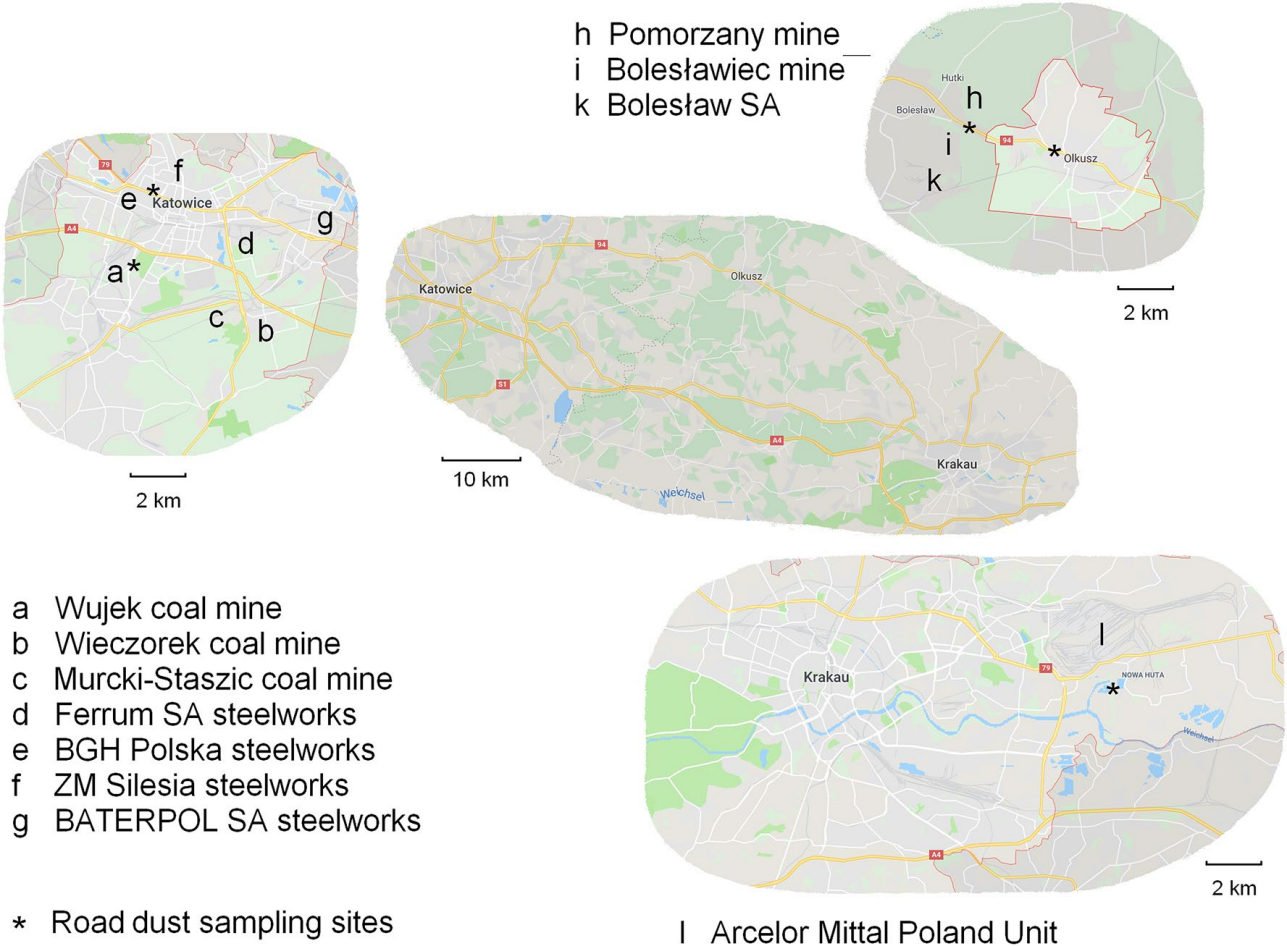
Sampling sites and major metallurgical plants and mining activities

In Olkusz, the road dust was collected from the city centre (Kościuszki Street, Górnicza Street, Szpitalna Street), from the vicinity of the Pomorzany mine (Kluczewska Street) and from around the closed Bolesławiec mine (Kolejowa Street). The dust samples from the different areas were mixed carefully before the dust was poured into two 10-L buckets. In Katowice, road dust was collected from roads in the vicinity of the Wujek mine (ul. Pola Street, Dziewięciu z wujka Street, and Gallusa Street) and in the city center (Mikołowska Street and Żeliwna Street). Mixing and filling of the dust samples from Katowice was performed in a similar way. In Krakow, the road dust sample was collected from discharge of the vacuum street sweeping vehicle after cleaning Ujastek Mogilski Street, Igołomska Street, Klasztorna Street, Żaglowa Street, and Podbagnie Street in the Nova Huta district. In the laboratory, the road dust samplers were dried at 110 °C for 24 h. Figure 1 shows the road dust sampling sites as well as major metallurgical plants and mining activities in this area.

#### Laboratory Procedure

**Fig. 2:**
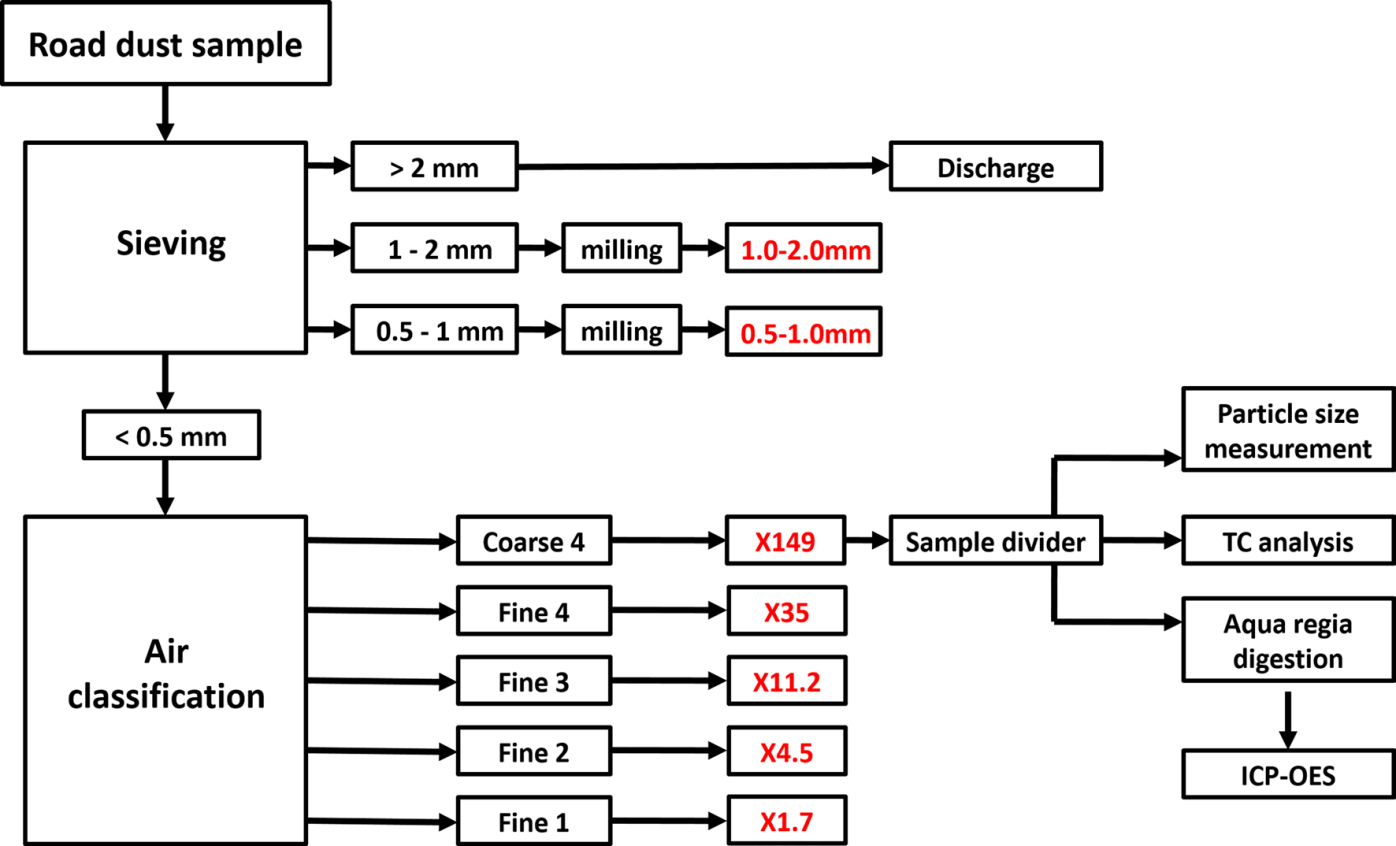
Sample preparation and analysis. The analytical procedure is shown exemplarily for a size fraction produced by air classification

The procedure of sample preparation and analysis is shown schematically in Fig. 2. In total, seven size fractions were produced for separate analysis by classification. In a first classification step the dry dust samples were sieved into four size fractions: < 0.5 mm, 0.5–1.0 mm, 1.0–2.0 mm, > 2.0 mm using a Fritsch Analysette 3 PRO vibratory sieve shaker. Subsequently, the size fraction < 0.5 mm was classified into five size fractions using a Hosokawa Alpine 100 MZR laboratory air classifier according to Lanzerstorfer (2015a). As reported in the literature (Lanzerstorfer 2015b), the concentrations of Cr and Ni are increased in air classified dust samples due to some erosion of classifier material. This makes evaluation of these metals in air classified samples impossible.

The quantity of the dust samples was reduced to a suitable volume for analysis using sample dividers, which were applied repeatedly (Haver & Boecker HAVER RT, Quantachrome Micro Riffler). The particle size distribution was determined using a Sympatec HELOS/RODOS laser diffraction instrument with dry sample dispersion. The calibration of the instrument was checked with a Sympatec SiC-P600’06 standard. The mass median diameter d_50_ of the size fractions was calculated assuming a constant particle density of the dust in all size fractions. For milling of the coarse size fractions before digestion a mixer mill (MM 301 from Retsch) was used.

For chemical analysis, 2 to 3 g dust samples were digested in 28 mL aqua regia under reflux for 2 h followed by appropriate dilution and filtration. Each sample was tested in duplicate. In the results the average values are presented. The concentrations of various metals were measured using a Horiba Jobin-Yvon Ultima 2 inductively coupled, plasma atomic emission spectroscopy system. For calibration, a MERCK CertiPur®ICP multi-element standard II was used. Details of the analytical methods can be found elsewhere (Lanzerstorfer 2015c).

The total carbon content (TC) was determined with a liquiTOC system from Elementar Analysensysteme. The system was calibrated using an Elementar Analysensysteme soil standard with 4.1% TOC/TC.

Method validation for ICP AES and TC was basically performed according ISO 17025, ISO 5275-1, and ISO guide 32 and 33. The statistic values linearity (*n* = 2 × 3), accuracy (trueness and precision *n* = 3 × 3), LOD (level of determination) and LOQ (level of quantification) from mean method standard deviation (*n* = 3 × 3) were determined. For TC, the evaluation of LOQ and LOD was not performed due to the samples magnitude higher TC content compared with the analytical limits of the used system. Method detection limit was calculated from the analytical detection limit in account with the sample digestion procedure and dilutions.

Microscopic images of particles from the five fine size fractions produced by air classification were made with a TESCAN MIRA 3 scanning electron microscope. Further information about the chemical composition of the particles was obtained in combination with energy dispersive X-ray spectroscopy (SEM-EDX). The increased C content measured in some samples in comparison to the TC measurements resulted from sample holder material, which was not covered by dust. The O content was increased for the same reason. However, the results can be corrected approximately using the results from the TC measurements under the assumption of Si, Al, and Fe being present as oxides and Ca and Mg being present as carbonates.

### Calculations

For assessment of the metal pollution of road dust and the probable contribution of anthropogenic sources, the enrichment factor (EF) can be used (Lu et al. 2010; Yuen et al. 2012). To minimize the variations caused by heterogeneous samples, these factors are usually normalized using a reference element (commonly used elements are Fe, Al, Ti, Mn). In Eq. (1), C_n_ is the mass concentration of the target component, whereas C_ref_ is the concentration of the selected reference component. B_n_ is the geochemical background concentration of the target component in the upper crust (Taylor and Mclennan 1995) and B_ref_ is the background concentration of the reference component:

1 $$ \left( {{\text{EF}} = {{\left( {\frac{{C_{n} }}{{C_{\text{ref}} }}} \right)} \mathord{\left/ {\vphantom {{\left( {\frac{{C_{n} }}{{C_{\text{ref}} }}} \right)} {\left( {\frac{{B_{n} }}{{B_{\text{ref}} }}} \right)}}} \right. \kern-0pt} {\left( {\frac{{B_{n} }}{{B_{\text{ref}} }}} \right)}}} \right) $$

The following ranges of EF were used for assessment of metal pollution: EF < 2: deficiency to minimal enrichment; EF = 2–5: moderate enrichment; EF = 5–20: significant enrichment; EF = 20–40: very high enrichment; and EF > 40: extremely high enrichment (Birch and Olmos 2008). Values of EF lower than 10 are considered to originate mostly from the background soil, whereas values higher than 10 are assumed to indicate anthropogenic sources of metal pollution (Liu et al. 2003). In this study, Mn was chosen as reference element as in a study on road dust by Hu et al. (2013).

To determine the contribution of the particle size fractions to the overall contamination of the road dust by a certain pollutant n, the grain size fraction loading GSFL can be calculated for each size fraction i (Sutherland et al. 2012):

2 $$ {\text{GSFL}}_{n,i} = \frac{{C_{n,i} \cdot x_{m,i} }}{{\sum\nolimits_{i = j}^{m} {C_{n,j} \cdot x_{m,j} } }} $$

In Eq. (2) *x*_m, i_ is the mass fraction of the size fraction i.

## Results and Discussion

Table 1 shows the validation parameters for the ICP AES analytic applied to the digested samples. It can be shown that the various elements have different quantitative and typical linear ranges according to their emission response. Some values from cadmium in the following result tables are below the limit of quantification but in the range of the limit of detection.

**Table 1:** Method parameters and statistic values ICP AES analysis according each element; linear range for analytical determination, mean relative standard deviation (RSD) from calibration; level of detection (LOD) and level of quantification (LOQ)

Element	Wavelength in (nm)	Linear range for analysis in (mg/L)	Mean RSD in (%)	LOD in (mg/kg)	LOQ in (mg/kg)
Fe	238.204	0.01–5	2.9	1.7	5.0
Al	396.152	0.05–10	4.6	1.7	5.0
Mn	257.610	0.005–0.5	3.8	0.2	0.5
As	189.042	0.025–2.5	4.7	1.7	5.0
B	208.959	0.05–5	1.9	0.2	0.5
Ba	455.403	0.025–2.5	2.3	0.3	1.0
Cd	228.802	0.001–1	2.0	0.2	0.5
Co	228.616	0.001–1	3.8	0.2	0.5
Cu	324.754	0.05–5	4.5	1.7	5.0
Pb	220.353	0.05–5	4.8	1.7	5.0
Sr	407.771	0.01–5	3.0	0.2	0.5
V	292.402	0.005–2.5	3.5	1.7	5.0
Zn	213.856	0.05–5	0.9	0.2	0.5

### Road Dust Composition

Table 2 shows the concentrations of various metals and other components in the entire road dust samples < 2.0 mm of the three towns calculated from the various size fractions and the respective mass fractions. The concentrations of the metals in the road dust from Krakow are within the reported range (Hwang et al. 2016), while in the road dust from Katowice the concentrations of Zn, Pb, As, and Cd were significantly increased. However, the concentrations are very similar to those reported for road dust from Katowice by Adamiec et al. (2016). Because metal emissions from coal mining are typically low (Lin et al. 2017; Tang et al. 2017), the probable reason for the increased concentrations could be the local steel industry. In contrast to the integrated steel mill in Krakow, which uses mainly iron ore as raw material base the steel mills in Katowice use scrap.

**Table 2:** Concentration of metals and other components in the road dust samples < 2.0 mm (in mg/kg of dry mass)

	Krakow	Katowice	Olkusz
TC	24,200	50,200	27,700
Fe	13,400	17,800	37,500
Al	3630	4840	3810
Mn	468	631	1700
As	11.0	32.6	96.1
B	24.1	15.3	33.5
Ba	51.0	88.9	87.9
Cd	0.4*	3.3	55.5
Co	5.1	5.9	11.0
Cu	352	84.8	414
Pb	17.8	207	957
Sr	37.2	48.0	51.1
V	26.5	31.3	30.7
Zn	142	868	9850

*Below LOQ

In the mining town Olkusz, the concentrations of Zn and Pb are greatly increased. Because concurrent emissions of Cd are typical for Zn ore processing (Dudka and Domy 1997), the extremely high Cd concentration can be explained. The high concentration of As is also because of the ore since As is a companion element (Gruszecka-Kosowska and Kicińska 2017).

Table 3 shows the enrichment factors calculated with Mn as the reference component and the average concentration of the components in the upper continental crust as background concentration (Taylor and Mclennan 1995). An extreme enrichment was found for Zn and Cd in the dust from Olkusz. Additionally, Pb and As were very highly enriched in this dust. In the dust from Katowice, the enrichment of these metals was somewhat less and the enrichment was even lower in the dust from Krakow. The enrichment of Cu was highest in Krakow (significant enrichment) and less in Olkusz and Katowice. For the other metals (Fe, Al, Ba, Sr, Co, and V), the EF showed a deficiency or only minimal enrichment.

**Table 3:**
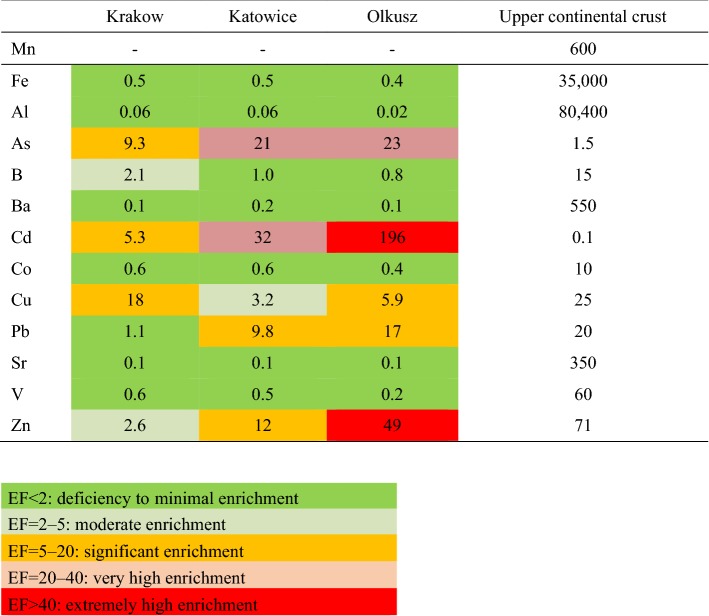
Enrichment factors EF for the different elements according to Eq. (1) based on road dust < 2.0 mm and the concentration of metals and other components in the upper continental crust (Taylor and Mclennan 1995) (in mg/kg of dry mass)

### Size Fractions

**Fig. 3:**
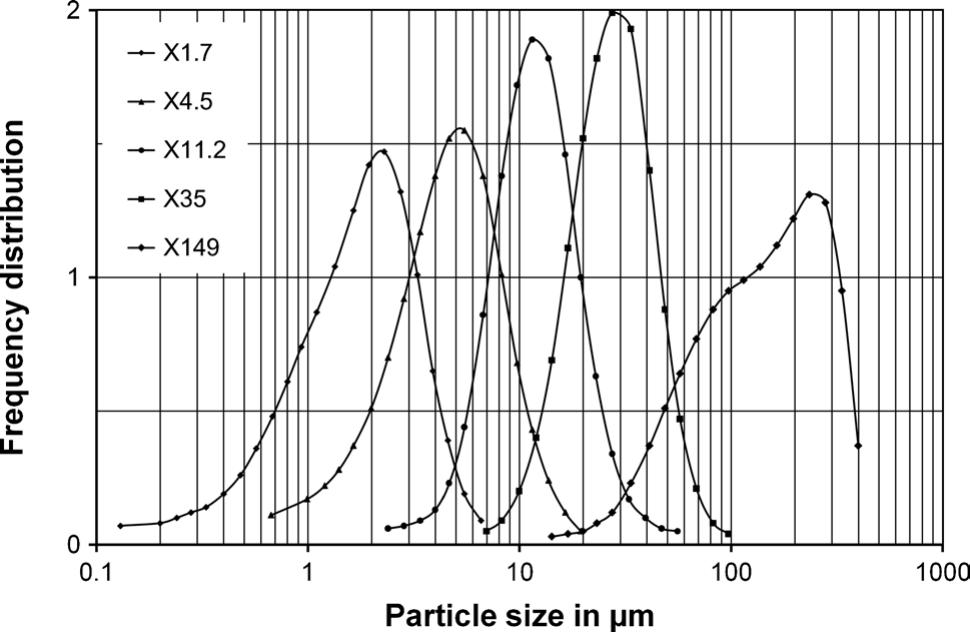
Size distribution of the size fractions of road dust from Katowice produced by air classification

Figure 3 shows the size distributions of the five size fractions produced from the Katowice road dust < 0.5 mm by air classification. Because the parameters in the air classification procedure were the same for all road dust samples, the size distributions were very similar. The mass median diameters were 1.7 ± 0.2 µm, 4.5 ± 0.4 µm, 11.2 ± 0.8 µm, 35 ± 10 µm, and 149 ± 18 µm for the five size fractions produced by air classification. In the following, these size fractions are named by their mass median diameters X1.7, X4.5, X11.2, X35, and X149. The mass fractions of the different size fractions produced by classification are summarized in Table 4. For the three finer size fractions, the values were quite similar for all three road dust samples. The mass fractions of the four coarser size fractions showed a much higher variation. While the two dust samples from the large cities, Krakow and Katowice, were quite similar in their size distribution, the road dust from Olkusz was characterized by an approximately fivefold occurrence of size fraction X35 combined with a reduced occurrence of the three coarsest size fractions.

**Table 4:** Mass fractions of the different size fractions produced by classification (in %)

	Air classification					Sieving	
	X1.7	X4.5	X11.2	X35	X149	0.5–1.0 mm	1.0–2.0 mm
Krakow	1.3	2.6	2.8	7.9	40.0	27.9	17.3
Katowice	1.5	3.1	3.7	7.0	46.8	26.3	11.7
Olkusz	1.4	3.4	5.0	37.2	28.0	18.3	6.9

In the road dust samples from Krakow and Katowice, X149 accounts for more than 40% of the total mass. Thus, the separation procedure might be improved by adding an additional sieve, for example a 200-µm sieve to the sieving stack to split this size fraction into two fractions.

In road dust from Thessaloniki, Greece, the size fractions 63–500 µm were the dominant (Bourliva et al. 2017). The corresponding size fraction X35 was also the dominant size fraction in the road dust from Katowice and Krakow. However, the mass fraction in this study was less, while the mass fraction > 500 µm was higher. The mass fractions X1.7 to X35 add up to a similar amount as the equivalent sieve fraction 0-63 µm in the study of Bourliva et al. (2017). In road dust from Beijing, China (Zhao et al. 2010), the mass distribution between the size fractions was quite similar; only the mass fraction of the finest mass fraction < 44 µm was less (approximately 4%), whereas in this study the corresponding size fraction (X1.7 to X11.2) summed up to 7–10%.

**Fig. 4:**
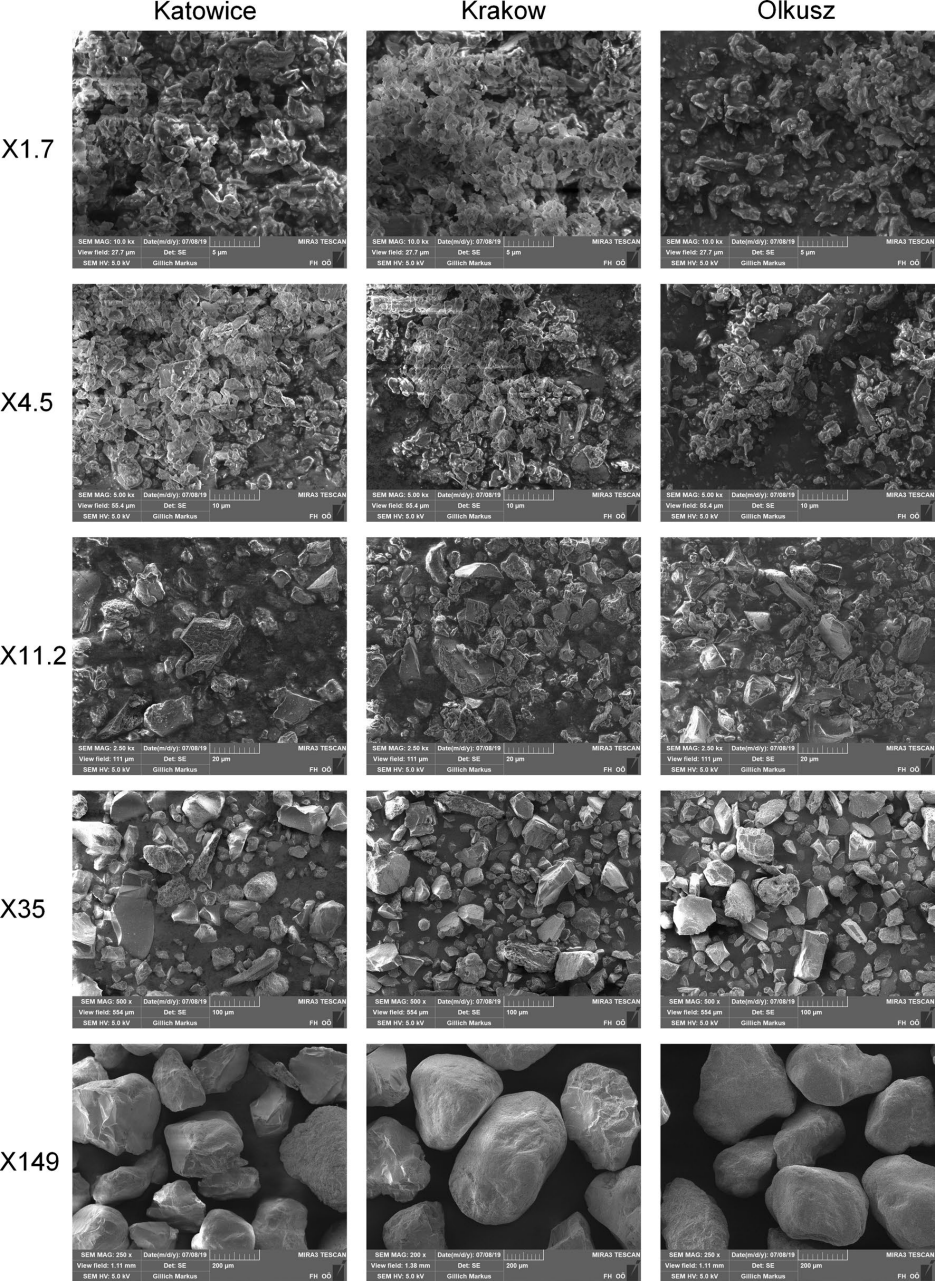
Microscopic images of particles of the five finest size fractions

Figure 4 shows microscopic images of the five finest size fractions produced by air classification for each road dust. The particles in the four finest size fractions are almost all edgy and often look like fragments. In contrast, the particles of the coarse fraction X149 were rather compact and partially rounded. No significant difference in particle shape was observed between the road dusts from the different locations.

### Size Dependence of the Metal Concentrations

**Fig. 5:**
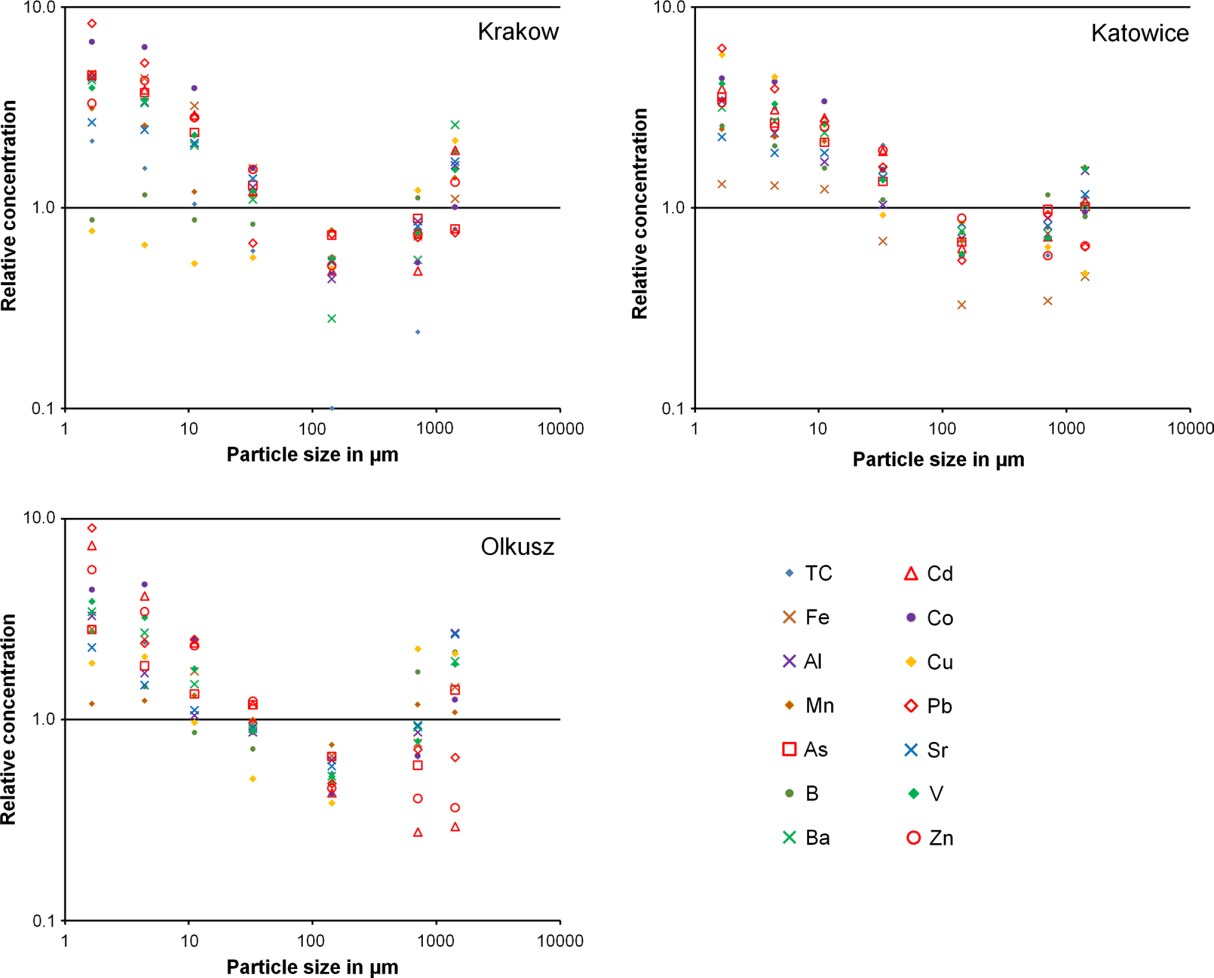
Relative concentration of various elements in the three road dusts

The composition of the road dust from Krakow, Katowice, and Olkusz by particle size is summarized in Table 5. With a few exceptions, the concentrations were highest in the finest size fraction. For many elements the minimum concentration is found in size fraction X149, while in the coarser size fractions 0.5–1.0 mm and 1.0–2.0 mm the concentrations were somewhat higher. Figure 5 shows the relative concentration of the various elements as a function of the particle size. For the size fractions produced by air classification, the mass median diameter was used as characteristic particle size. For the two coarse size fractions, the characteristic particle size was calculated as the geometric mean of the upper and lower sieve size. The maximum relative concentration was approximately 10:1 in relation to the concentration in the whole road dust < 2.0 mm and the minimum relative concentration was approximately 0.1:1. In Fig. 5, the concentration minima for most of the elements in the range of X149 are clearly visible.

**Table 5:** (a) Composition of Krakow road dust by particle size, (b) composition of Katowice road dust by particle size and (c) composition of Olkusz road dust by particle size

		X1.7	X4.5	X11.2	X35	X149	0.5–1.0 mm	1.0–2.0 mm
*(a)*								
TC	g/kg	134	98	65	38	6.1	15	49
Fe	g/kg	62	59	43	21	7.3	9.6	14.8
Al	g/kg	16.2	12.2	7.6	4.6	1.6	3.1	5.9
Mn	mg/kg	1470	1210	565	543	364	364	663
As	mg/kg	50	41	26	14.2	5.0	9.7	8.6
B	mg/kg	21	28	21	20	13.7	27	46
Ba	mg/kg	220	169	104	56	14.3	28	132
Cd	mg/kg	1.9	1.6	1.2	0.5	0.2*	0.2*	0.8
Co	mg/kg	34	32	20	8.0	2.4	2.7	5.1
Cu	mg/kg	270	230	186	199	176	430	760
Pb	mg/kg	148	94	50	11.9	13.2	12.7	13.4
Sr	mg/kg	99	91	78	52	19.9	30	63
V	mg/kg	105	92	61	32	14.6	20	41
Zn	mg/kg	470	610	400	220	73	105	190
*(b)*								
TC	g/kg	176	158	133	103	35	29	56
Fe	g/kg	52	51	49	27	13.0	13.6	18.0
Al	g/kg	16.5	11.4	8.2	5.0	3.4	4.3	7.4
Mn	mg/kg	1550	1420	1350	883	428	501	1000
As	mg/kg	116	86	69	44	22	32	33
B	mg/kg	39	31	24	16.7	11.5	17.7	13.8
Ba	mg/kg	280	240	210	138	68	66	91
Cd	mg/kg	13.1	10.3	9.4	6.4	2.1	2.4	3.6
Co	mg/kg	26	25	20	9.1	3.4	4.2	5.6
Cu	mg/kg	490	380	220	78	71	54	40
Pb	mg/kg	1290	810	560	330	113	191	132
Sr	mg/kg	108	90	90	68	40	39	56
V	mg/kg	130	103	82	43	18.4	22	49
Zn	mg/kg	2900	2200	2200	1680	770	500	560
*(c)*								
TC	g/kg	95	67	49	27	15	18	59
Fe	g/kg	107	95	66	36	25	29	55
Al	g/kg	12.5	6.5	4.0	3.3	2.4	3.3	10.3
Mn	mg/kg	2030	2100	2230	1690	1270	2010	1840
As	mg/kg	270	178	129	114	63	57	135
B	mg/kg	92	49	29	24	16.3	58	73
Ba	mg/kg	303	238	132	79	46	81	172
Cd	mg/kg	410	230	134	66	24	15.3	16.3
Co	mg/kg	49	52	28	9.6	4.7	7.3	13.9
Cu	mg/kg	790	850	400	210	159	930	880
Pb	mg/kg	8600	2300	2400	920	460	680	620
Sr	mg/kg	117	76	57	47	30	48	136
V	mg/kg	119	99	55	27	16	24	58
Zn	mg/kg	55,000	34,000	23,000	12,200	4500	4000	3600

*Below LOQ

The road dust from Krakow is generally only slightly polluted. Nevertheless, in the finest size fractions X1.7 and X4.5, which are most relevant for resuspension into the air, the concentrations of several metals are markedly increased. A different behaviour was observed for Cu. A slight decrease from size fraction X1.7 to size fraction X149 was found. However, the highest concentrations were found in the coarse fractions 0.5–1.0 mm and 1.0–2.0 mm.

In the road dust size fractions from Katowice, the concentrations of the metals Co, Mn, and V as well as of the metals Al and Fe were similar to Krakow. In contrast, for Cd, Pb, and Zn the concentrations were approximately ten times higher.

In the road dust from Olkusz, the generally extremely high concentrations of Fe and the metals Zn, Cd, As, and Pb result from the high concentrations in the soil in this area (Gruszecka-Kosowska and Kicińska 2017). The high concentrations in the coarse size fractions might be attributed to the local soil as well as to mining and ore transport activities, whereas the extreme concentrations in the finest size fractions presumably originate mainly from metallurgical activities, because such fine particles are typical for metallurgical fumes (Tossavainen 1976). In the road dust from Olkusz, the size dependence of the concentrations of Zn, Cd, and Pb is even more pronounced than in the dusts from the two larger towns. The concentration of other elements, for example Al, Ba, Sr, and V, was in the same range as in the two other road dust samples.

**Fig. 6:**
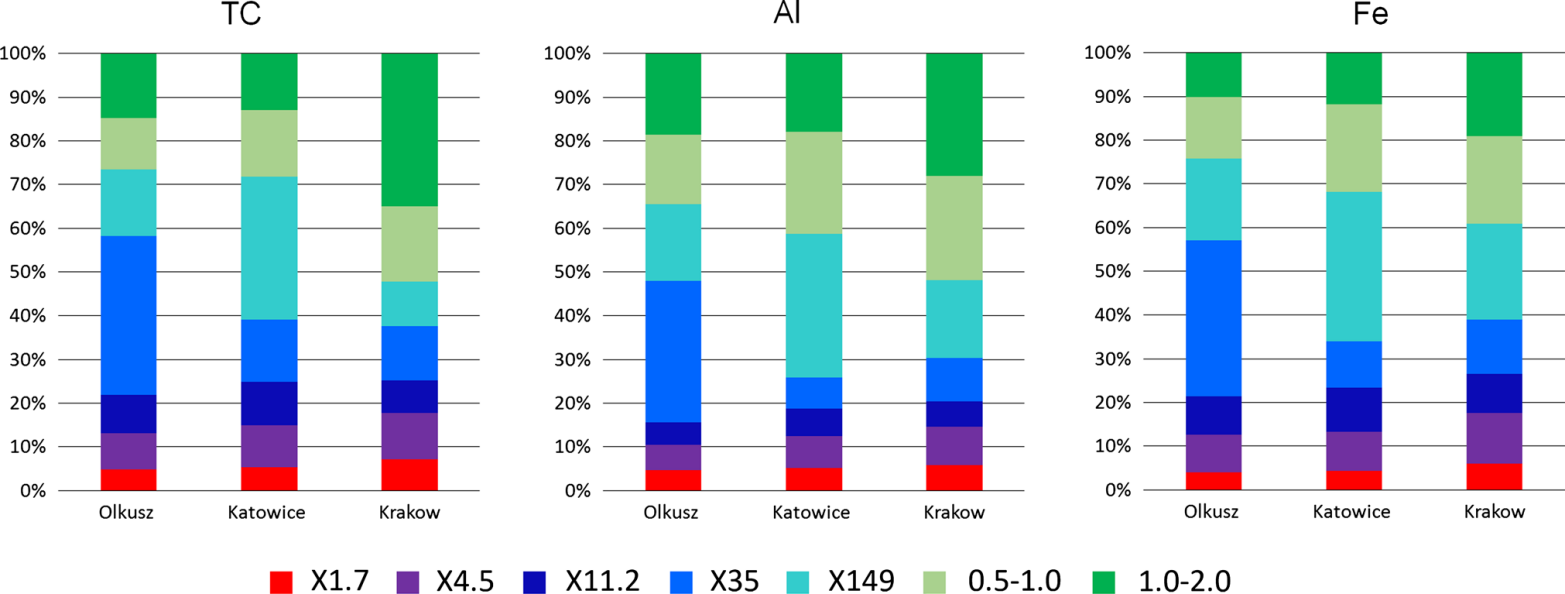
Distribution of the mass of TC, Al and Fe within the size fractions

**Fig. 7:**
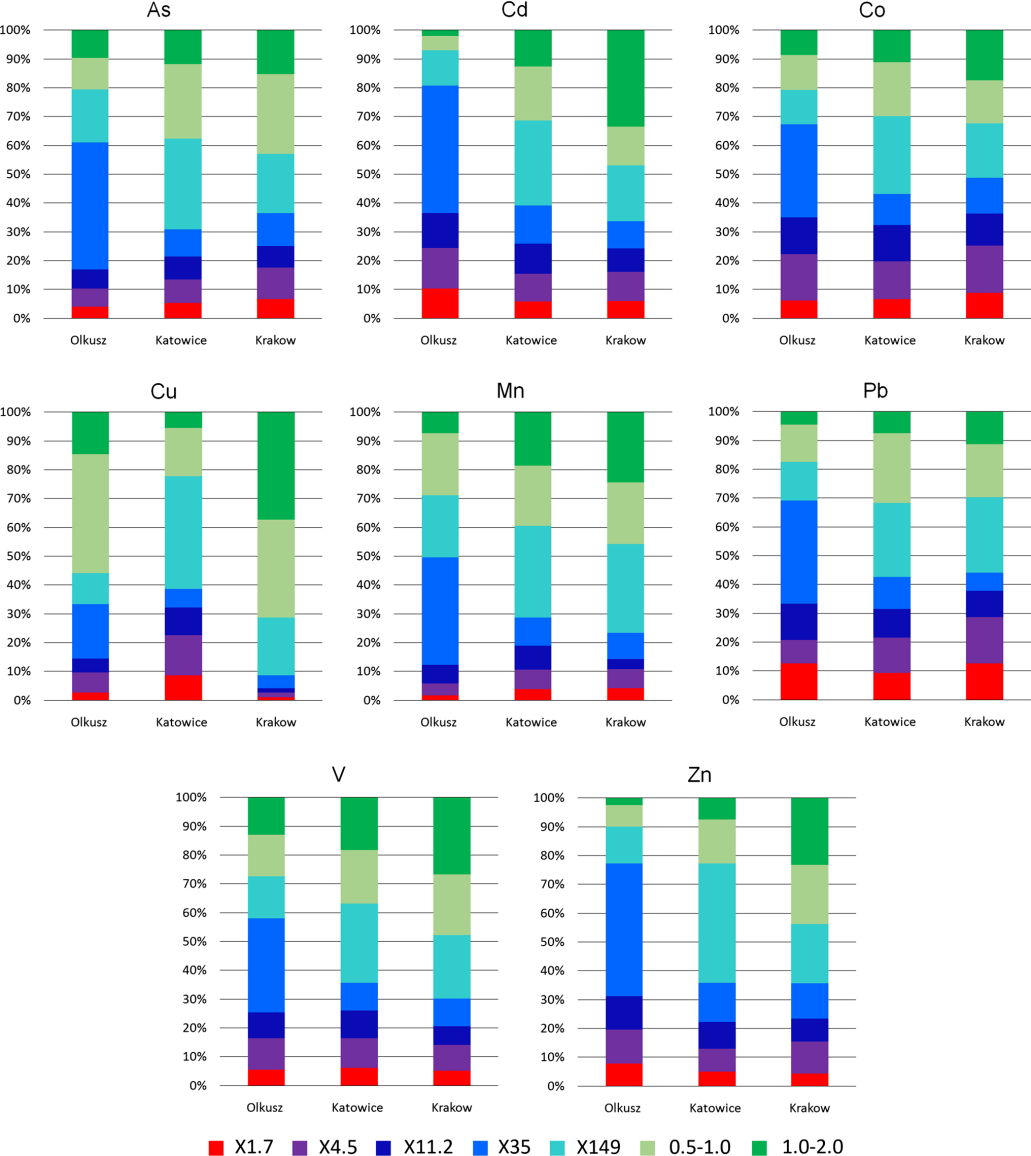
Distribution of the mass of various metals within the size fractions

In Figs. 6 and 7, the contributions of the particle size fractions to the overall contamination calculated by Eq. (2) are shown. The fraction of the elements contained in X1.7 and X4.5 is shown in red and violet. These size fractions are especially relevant with respect to resuspension into the air. The sum of the colours red, violet, dark blue, blue, and maybe half of light blue represents the mass fraction of the elements in the particle size fractions of road dust X1.7 to X35 more easily removed by wash-off during rainfall. The two green colours and half of the light blue represent the mass fraction of the elements contained in the coarse dust, which is less mobile.

The mass fraction of most components in the easily resuspended size fractions was similar for all three dusts. For TC, Al, and Fe, it was in the range of approximately 10–15% (Fig. 5). For the metals As and V, the picture was similar. For Cd, Co, Pb, and Zn, the fraction in easy to resuspend size fractions was higher (approximately 15–25%), and for Mn, it was lower (approximately 5–10%). For Cu, the variation between the three road dusts was substantially higher. While more than 20% of the Cu was in the two finest size fractions of the road dust from Katowice, it was less than 10% for Olkusz and as little as 2.7% for Krakow.

For the fraction of the components contained in the easy to wash-off size fractions, the variation was considerably higher. However, with the exception of Cu there was a general trend of declining mass fraction from the Olkusz dust to the Katowice dust and further to the Krakow dust. A similar pattern (approximately 60%, 50%, and 40%) was found for TC, Al, Fe, Mn, and V. Other patterns were 70%, 50%, and 50% (for As and Co) and 80%, 50%, and 50% (Cd, Pb, and Zn). A totally different result was found for Cu where the easy to wash-off mass fraction was 40%, 60%, and 20% for the dusts from Olkusz, Katowice, and Krakow, respectively.

**Fig. 8:**
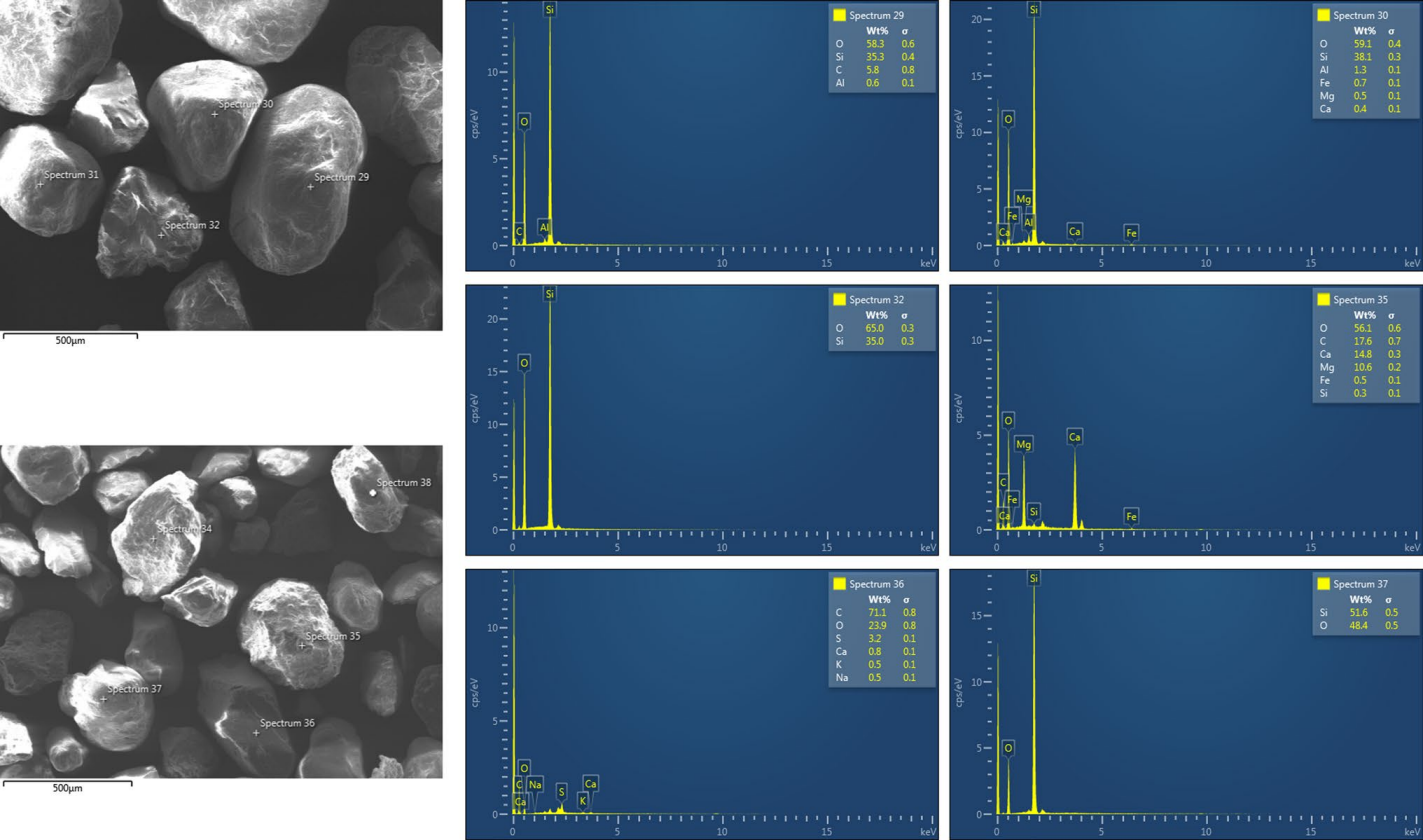
SEM-EDX results for particles from the size fraction X149 from Krakow (upper picture) and Katowice (lower picture)

The SEM-EDX results of the road dust samples show an increasing content of Si with increasing particle size. In the finest size fraction, X1.7, the corrected Si concentration was 11–18%, whereas in the size fraction X149 it was 34–40%. Figure 8 shows SEM-EDX results for particles from size fraction X149. In most particles, a high Si content was found. Therefore, they are silicates (spectrum 29 and 30) or even SiO_2_ (spectrum 32 and 37). The particle with the spectrum 35 contains almost no Si. Because of its Ca, Mg, C, and O content, it could be mainly dolomite. The particle with the spectrum 36 consists mainly of C. Because it contains also some S, it could be a rubber particle.

## Conclusions

A new combined classification method for sample preparation in road dust studies has been applied in a study of the size-dependence of the metal concentration of road dust in three Polish metallurgical and mining industry towns. Thereby, air classification of the samples was applied additionally to sieving to cover the particle size range from approximately 2 µm to 2 mm.

The concentration of metals in the road dust from Krakow, Katowice, and Olkusz varied, especially with respect to the concentration of As, Cd, Pb, and Zn. Extreme concentrations were found in the road dust from the Pb and Zn mining town Olkusz. The high concentrations of Pb and Zn in the coarse size fractions might be attributed to the local soil and to mining and ore transport activities. The extreme concentrations in the finest size fractions presumably originate mainly from fumes from metallurgical activities. In Katowice, the concentrations of the metals were high, whereas in Krakow, the concentrations were within a typical range.

The mass fraction of the fine size fractions was in the low percentage range. The distribution of the metals in the different size fractions of road dust is not evenly distributed but shows a significant enrichment up to 10:1 in the finest size fraction. This size dependence has important effects. First, the least polluted size fraction was generally the fraction 200–500 µm, whereas the larger fractions were more heavily loaded with metals. Second, investigation of different size fractions of the road dust instead of the investigation of whole road dust samples provides much more information, especially for the evaluation of the transfer of components from the road dust into the atmosphere or to the run-off during rainfall. Third, the highest concentrations were measured in the finest size fraction < 2 µm. Thus, size separation by sieving is not sufficient because of the size limitations of sieving.

The strong size dependence of the concentration of several metals confirms the importance of the new air classification step added to the conventional sieving procedure. Thus, the metal concentrations in the size fractions most relevant with respect to resuspension into the air can be determined more exactly. The new classification procedure might further be improved by adding a 200-µm sieve to the sieving stack to split the largest size fraction X149, which accounts for up to 46% of the total road dust samples into two fractions.
